# Efficacy of anti-hyperkalemic agents during cardiopulmonary resuscitation in out-of-hospital cardiac arrest

**DOI:** 10.1016/j.heliyon.2024.e36345

**Published:** 2024-08-15

**Authors:** Gun Tak Lee, Daun Jeong, Jong Eun Park, Se Uk Lee, Taerim Kim, Hee Yoon, Won Chul Cha, Min Seob Sim, Ik Joon Jo, Sung Yeon Hwang, Tae Gun Shin

**Affiliations:** a Department of Emergency Medicine, Samsung Medical Center, Sungkyunkwan University School of Medicine, Seoul, Republic of Korea; b Department of Emergency Medicine, College of Medicine, Kangwon National University, Chuncheon, Gangwon-do, Republic of Korea

**Keywords:** Hyperkalemia, Cardiac arrest, Calcium, Sodium bicarbonate

## Abstract

**Aim:**

We assessed the efficacy of anti-hyperkalemic agents for alleviating hyperkalemia and improving clinical outcomes in patients with out-of-hospital cardiac arrest (OHCA).

**Methods:**

This was a single-center, retrospective observational study of OHCA patients treated at tertiary hospitals between 2010 and 2020. Adult patients aged 18 or older who were in cardiac arrest at the time of arrival and had records of potassium levels measured during cardiac arrest were included. A linear regression model was used to evaluate the relationship between changes in potassium levels and use of anti-hyperkalemic medications. Cox proportional hazards regression analysis was performed to analyze the relationship between the use of anti-hyperkalemic agents and the achievement of return of spontaneous circulation (ROSC).

**Results:**

Among 839 episodes, 465 patients received anti-hyperkalemic medication before ROSC. The rate of ROSC was higher in the no anti-hyperkalemic group than in the anti-hyperkalemic group (55.9 % vs 47.7 %, P = 0.019). The decrease in potassium level in the anti-hyperkalemic group from pre-ROSC to post-ROSC was significantly greater than that in the no anti-hyperkalemic group (coefficient 0.38, 95 % confidence interval [CI], 0.13–0.64, P = 0.003). In Cox proportional hazards regression analysis, the use of anti-hyperkalemic medication was related to a decreased ROSC rate in the overall group (adjusted hazard ratio [aHR] 0.66, 95 % CI, 0.54–0.81, P < 0.001), but there were no differences among subgroups classified according to initial potassium levels.

**Conclusions:**

Anti-hyperkalemic agents were associated with substantial decreases in potassium levels in OHCA patients. However, administration of anti-hyperkalemic agents did not affect the achievement of ROSC.

## Abbreviations

OHCA: (out-of-hospital cardiac arrest)
CPR: (cardiopulmonary resuscitation)
ECG: (electrocardiogram)
ED: (emergency department)
IHD: (ischemic heart disease)
CKD: (chronic kidney disease)
ROSC: (return of spontaneous circulation)
IQR: (interquartile range)
HR: (hazard ratio)
CI: (confidence interval)

## 1 Introduction

Globally, out-of-hospital cardiac arrest (OHCA) is a significant public health problem with a poor prognosis [[1], [2], [3]]. Despite advancements in resuscitation care, the likelihood of survival for OHCA patients with a favorable neurological outcome are still low [[1], [2], [3], [4], [5]]. To improve the prognosis of patients with OHCA, international guidelines recommend investigating and treating the etiology of reversible cardiac arrest in addition to high-quality cardiopulmonary resuscitation (CPR) [6,7].

Hyperkalemia is a hazardous electrolyte imbalance that can result in cardiac arrest by producing deadly arrhythmias [[8], [9], [10], [11]]. Potassium is the principal intracellular cation, with approximately 98 % of it distributed within the intracellular space [12,13]. The allocation of total body potassium between the intracellular and extracellular spaces establishes a notable potassium concentration gradient between these two compartments. The electrical characteristics of cells can be significantly changed by even minor adjustments in the smaller extracellular concentration of potassium, due to the dependence of membrane resting potential on the ratio of intracellular and extracellular potassium concentrations [14]. Hyperkalemia is hazardous because it can cause depolarization in cell membranes that are electrically active, including cardiac and skeletal muscle [15,16]. The myocardium is highly sensitive to changes in potassium concentration. Imbalances in the potassium concentration gradient during hyperkalemia can lead to a series of abnormalities in the ECG [17]. Early electrocardiographic indicators include peaked T waves on the ECG followed by flattened or absent T waves, a longer PR interval, a widened QRS complex, deepened S waves, and the merging of S and T waves. As hyperkalemia continues, the ECG can develop a sine-wave pattern, and the patient can go into cardiac arrest [10,18,19]. Several interventions have been proposed for life-threatening hyperkalemia, including intravenous calcium, bicarbonate, and insulin with glucose [[6], [7], [8],^20^]. It remains unclear, however, if such interventions provided during CPR effectively reduce serum potassium levels and thereby improve the patient's prognosis [[21], [22], [23], [24], [25], [26]]. This study's objective was to assess the efficacy of anti-hyperkalemic medicines in treating hyperkalemia and improving clinical outcomes.

## 2 Methods

### 2.1 Study design and population

This was a retrospective, observational study of OHCA patients treated at Samsung Medical Center (a 1960-bed, university-affiliated, tertiary referral hospital with an annual census of 70,000 located in Seoul, South Korea), from January 2010 to December 2020. The primary analysis included adult patients who experienced OHCA and were 18 years of age or older. Both shockable and non-shockable initial ECG rhythms were included in the analysis. Excluded from the study were cases in which patients achieved return of spontaneous circulation (ROSC) before reaching the hospital and were not in cardiac arrest upon arrival, as well as cases in which CPR was not performed at the hospital due to patients having limitations on resuscitation, such as a do-not-resuscitate order. In addition, cases whose potassium concentration was not measured during CPR were also excluded. This study was approved by the Institutional Review Board of Samsung Medical Center (IRB No.: 2022-04-130). The need for informed consent was waived given the study's retrospective, observational, and anonymous nature.

### 2.2 Data collection and outcome measures

The following variables were extracted from the institutional cardiac arrest registry and electronic medical records: age, sex, preexisting conditions including hypertension, diabetes, chronic pulmonary disease, ischemic heart disease (IHD), malignancy, and chronic kidney disease (CKD), initial ECG rhythm, witnessed cardiac arrest, bystander-provided CPR, arrest location, laboratory test results including pH, HCO_3_, and potassium levels, medications including sodium bicarbonate, calcium gluconate, insulin with glucose, and epinephrine, ROSC, one-month survival, and three-month survival. To investigate changes in potassium level during CPR, the initial potassium level during CPR, subsequent potassium levels during CPR, and potassium levels after ROSC were compared. In this study, ROSC was defined as palpable pulse at any time. In the event of multiple cardiac arrests, ROSC was defined as the first palpable pulse. Changes in potassium level (delta potassium) were also compared between each measurement time point. When potassium levels were measured multiple times during CPR, the first value measured after the initial measurement was used for analysis; this is referred to as the subsequent level. The interval to potassium concentration measurement was defined as the time between the patient's arrival at the ED and the measurement report. Electronic medical data were extracted from the Clinical Data Warehouse DARWIN-C of Samsung Medical Center for this study. We analyzed anonymized clinical data and performed no manipulations on the original data other than statistical analysis.

The anti-hyperkalemic group comprised individuals who received any potassium lowering agents such as sodium bicarbonate, calcium, or insulin with glucose, whereas the no anti-hyperkalemic group comprised individuals who did not receive any of the medications listed above. Aside from the American Heart Association (AHA) recommendation, no institute-specific protocol was used to treat hyperkalemia in patients with cardiac arrest; drug administration was left to the discretion of the clinician. The primary outcome of this study was the ROSC rate.

### 2.3 Statistical analysis

Baseline characteristics of groups are reported as median values and interquartile ranges (IQRs) for continuous variables and as numbers and percentages for categorical variables. Groups were compared using Wilcoxon rank-sum tests for continuous variables and chi-squared tests for categorical variables. The Wilcoxon signed-rank test was used to compare repeated measured potassium levels. The relationship between delta potassium level and use of anti-hyperkalemic medication was evaluated using a linear regression model. The log-rank test was used to compare Kaplan-Meier curves for the achievement of ROSC according to the use of anti-hyperkalemic medication. The relationship between ROSC and usage of anti-hyperkalemic medication in the presence of moderate to severe hyperkalemia was assessed using Cox proportional hazards regression analysis and results are expressed as hazard ratios (HRs) and 95 % confidence intervals (CIs). Moderate to severe hyperkalemia was defined as potassium levels of 6.5 mmol/L or higher. Adjustments were made for variables with significant differences between groups and variables known to be prognostic factors. Age, gender, diabetes, malignancy, CKD, chronic pulmonary disease, arrest location, bystander CPR, bystander witnessed, initial ECG rhythm, and initial potassium level were the final variables included. In addition, a subgroup analysis was performed for individuals with potassium levels of 6.5 mmol/L or higher, 7.5 mmol/L or higher, and 8.5 mmol/L or higher to validate the effects of anti-hyperkalemic agents based on the degree of hyperkalemia. The predicted level of potassium during CPR is presented as a 95 % CI and was generated using a quadratic prediction plot referencing all potassium levels measured during CPR. P-values less than 0.05 were considered statistically significant. STATA software (STATA Corporation, College Station, TX, USA) version 17.0 was used to analyze the data.

## 3 Results

### 3.1 Baseline characteristics

In this study, we assessed the eligibility of 1370 OHCA patients. Among them, 66 patients were not in cardiac arrest upon arrival at the ED, and in 465 patients, serum potassium levels were not measured during CPR. After excluding these cases, 839 OHCA cases were analyzed. The anti-hyperkalemic treatment group comprised 465 cases while the no anti-hyperkalemic group comprised 374 cases. Calcium was the most commonly used drug among the 465 individuals in the anti-hyperkalemic group (N = 369/465, 79.4 %), followed by sodium bicarbonate (N = 321/465, 69.0 %) and insulin (N = 64/465, 13.8 %).

Table 1 shows the baseline characteristics of the study population. The median age of the study population was 68 years, and 65.1 % of the participants were male (n = 546). There were no statistically significant differences in age (no anti-hyperkalemic vs anti-hyperkalemic: 67 years [IQR 53–78] vs 68 years [IQR 53–80], P = 0.275) or gender (no anti-hyperkalemic vs anti-hyperkalemic: 63.6 % vs 66.2 %, P = 0.432) between the two groups. Diabetes (24.3 % vs 16.3 %, P = 0.005), chronic kidney disease (8.6 % vs 2.9 %, P = 0.001), and malignancy (18.5 % vs 11.8 %, P = 0.007) were more prevalent in the anti-hyperkalemic group than in the no anti-hyperkalemic group. In contrast, the prevalence of chronic pulmonary disease was higher in the no anti-hyperkalemic group than in the anti-hyperkalemic group (4 % vs 1.7 %, P = 0.043). Cardiac arrest occurred more frequently in private places in the anti-hyperkalemic group than in the no anti-hyperkalemic group (56.5 % vs 64.6 %, P = 0.018).

**Table 1:** Baseline characteristics of all patients.

	Overall (n = 839)	No anti-hyperkalemic (n = 374)	Anti-hyperkalemic (n = 465)	P
Age, years	68 (53–79)	67 (53–78)	68 (53–80)	0.275
Male sex, no. (%)	546 (65.1 %)	238 (63.6 %)	308 (66.2 %)	0.432
Preexisting conditions, no. (%)				
Hypertension	270 (32.2 %)	119 (31.8 %)	151 (32.5 %)	0.840
Diabetes	174 (20.7 %)	61 (16.3 %)	113 (24.3 %)	0.005
Chronic pulmonary disease	23 (2.7 %)	15 (4.0 %)	8 (1.7 %)	0.043
IHD	68 (8.1 %)	28 (7.5 %)	40 (8.6 %)	0.556
Malignancy	130 (15.5 %)	44 (11.8 %)	86 (18.5 %)	0.007
CKD	51 (6.1 %)	11 (2.9 %)	40 (8.6 %)	0.001
CVA	68 (8.1 %)	28 (7.5 %)	40 (8.6 %)	0.556
Arrest location, no. (%)				0.018
Private place	504 (60.9 %)	209 (56.5 %)	295 (64.6 %)	
Public place	323 (39.1 %)	161 (43.5 %)	162 (35.5 %)	
Bystander witnessed, no. (%)	574 (70.6 %)	271 (73.8 %)	303 (69.4 %)	0.066
Bystander CPR, no. (%)	365 (44.3 %)	165 (45.2 %)	200 (43.6 %)	0.639
Initial ECG rhythm, no. (%)				
Shockable	65 (7.8 %)	26 (7.0 %)	39 (8.4 %)	0.441
Non-shockable	772 (92.2 %)	347 (93.0 %)	425 (91.6 %)	0.544
Initial pH level	6.92 (6.80–7.04)	6.96 (6.84–7.08)	6.89 (6.79–6.99)	<0.001
Epinephrine use, no. (%)	820 (97.7 %)	355 (94.9 %)	465 (100.0 %)	<0.001

IHD, ischemic heart disease; CKD, chronic kidney disease; CVA, cerebrovascular accident; CPR, cardiopulmonary resuscitation; ECG, electrocardiogram.

### 3.2 Comparisons of potassium levels

**Fig. 1:**
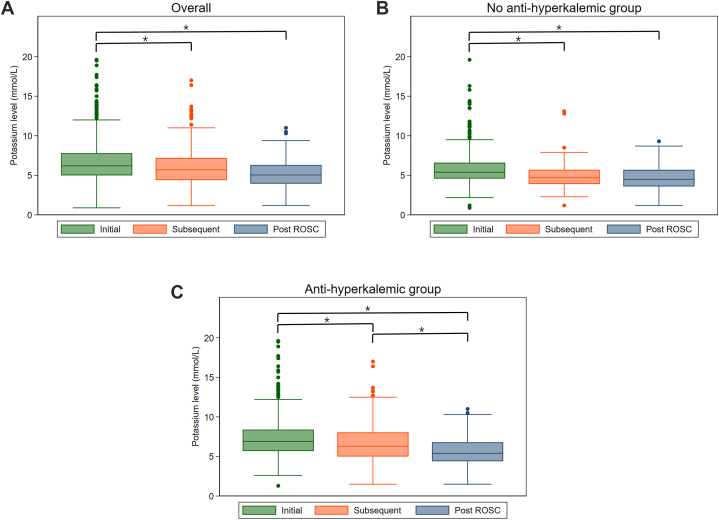
Initial (A), subsequent (B), and post ROSC (C) potassium levels (*P < 0.05). Initial refers to the first measured value during CPR. Subsequent refers to the value that is measured during CPR after the initial measurement. Post ROSC refers to the first measured value after ROSC.

Initial and subsequent potassium levels during CPR were measured at 7 (IQR 3–9) and 16 (IQR 11–20) minutes following the initiation of CPR (Fig. 1, Supplementary Fig. 1 and Supplementary Table A). Potassium levels were highest initially (6.2 mmol/L, IQR 5.0–7.8) and decreased significantly subsequently and post-ROSC (5.7 mmol/L, IQR 4.4–7.2; 5.1 mmol/L, IQR 4.0–6.3) (Fig. 1A & Supplementary Table A). Subsequent potassium levels were significantly lower than initial levels in both groups. In the anti-hyperkalemic group, post-ROSC levels were significantly lower than subsequent levels (P = 0.02), while post-ROSC levels were no different from subsequent levels in the no anti-hyperkalemic group (P = 0.60) (Fig. 1B and C).The anti-hyperkalemic group had significantly higher initial (6.9 mmol/L [IQR 5.7–8.4] vs 5.4 mmol/L [IQR 4.6–6.6], P < 0.001), subsequent (6.3 mmol/L [IQR 5.0–8.0] vs 4.7 mmol/L [IQR 3.9–5.7], P < 0.001), and post-ROSC (5.4 mmol/L [IQR 4.4–6.8] vs 4.5 mmol/L [IQR 3.6–5.7], P < 0.001) potassium levels than the no anti-hyperkalemic group.

When delta potassium levels were compared, potassium level variation from the initial measurement to the post-ROSC measurement was significantly greater in the anti-hyperkalemic group than the no anti-hyperkalemic group (coefficient 0.38, 95 % CI, 0.13–0.64, P = 0.003). However, there was no discernible difference between the two groups in the changes in potassium levels from initial to subsequent and from subsequent to post-ROSC time periods (coefficient 0.21, 95 % CI, −0.14–0.56, P = 0.234) (Supplementary Table B).

**Fig. 2:**
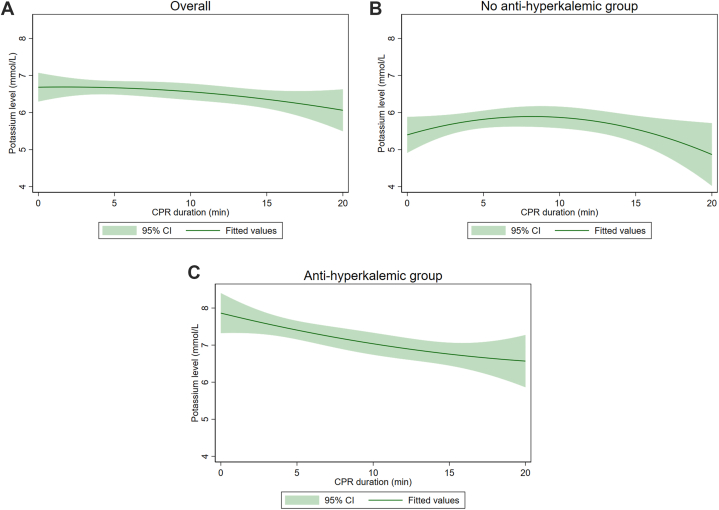
Fitted curve of the predictive potassium level for overall (A), no-anti hyperkalemic group (B) and anti-hyperkalemic group (C). A quadratic prediction plot was generated to visualize the relationship between potassium levels and ROSC, incorporating all potassium levels measured during CPR.

Predicted potassium levels during CPR are shown in Fig. 2. Overall, while CPR was continued, there was a decrease in the concentration of potassium (Fig. 2A). The potassium level in the no anti-hyperkalemic group appeared to rise steadily for the first 10 min of CPR, and then began to decrease (Fig. 2B). In contrast, the potassium level in the anti-hyperkalemic group showed a consistent decline throughout CPR (Fig. 2C).

### 3.3 Outcomes

The rate of ROSC was higher in the no anti-hyperkalemic group than in the anti-hyperkalemic group (55.9 % vs 47.7 %, P = 0.019). The interval to ROSC was longer in the anti-hyperkalemic group (7 min [IQR 5–11] vs 10 min [IQR 7–16], P < 0.001). In addition, in the no anti-hyperkalemic group, survival to ER discharge was significantly higher (34.2 % vs 22.3 %, P < 0.001) than in the anti-hyperkalemic group. However, neither 3-month survival nor favorable neurology differed significantly between groups (Table 2).

**Table 2:** Outcomes.

	Overall (n = 839)	No anti-hyperkalemic (n = 374)	Anti-hyperkalemic (n = 465)	P
ROSC	408 (48.6 %)	209 (55.9 %)	222 (47.7 %)	0.019
Time to ROSC^a^, min	9 (6–11)	7 (5–11)	10 (7–16)	<0.001
Survival to ER discharge	227 (27.1 %)	128 (34.2 %)	99 (22.3 %)	<0.001
3-month survival	29 (3.5 %)	17 (4.6 %)	12 (2.6 %)	0.121
3-month survival with favorable neurologic outcome	25 (3.0 %)	14 (3.7 %)	11 (2.4 %)	0.243

a Duration between the initiation of advanced cardiovascular life support and the restoration of spontaneous circulation (ROSC).

**Fig. 3:**
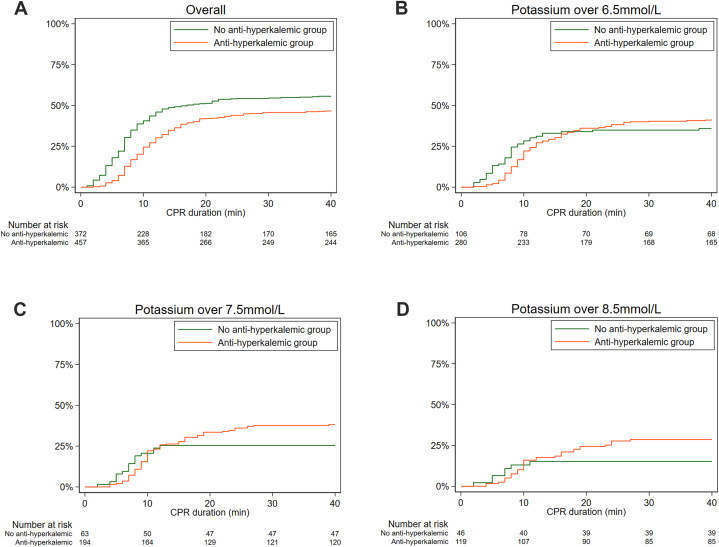
Kaplan-Meier plot depicting proportion of ROSC according to initial potassium level.

The rates of achievement of ROSC over time for the overall group and for groups stratified by initial potassium level are depicted in the Kaplan Meier plot in Fig. 3. The overall no anti-hyperkalemic group had a higher ROSC rate (log-rank test, P < 0.001) (Fig. 3A), whereas the anti-hyperkalemic group with a high initial potassium level had a higher ROSC rate in subgroup analysis, but none of the subgroup results were significant (Fig. 3B–D).

Findings of univariable and multivariable Cox regression analyses to evaluate the association between ROSC and the administration of anti-hyperkalemic drugs based on a specific initial potassium level are shown in Table 3. The use of anti-hyperkalemic medications was not associated with ROSC rate in any subgroup (all P > 0.05). In the subgroup analysis involving individuals with moderate to severe hyperkalemia, there was a significant correlation between witnessed cardiac arrest and the achievement of ROSC in the group with potassium levels above 6.5 mmol/L (adjusted hazard ratio [aHR] 2.17, 95 % CI, 1.46–3.22, P < 0.001) and above 7.5 mmol/L (adjusted hazard ratio [aHR] 2.40, 95 % CI, 1.42–4.06, P = 0.001). However, this correlation was not significant in the group with potassium levels above 8.5 mmol/L (adjusted hazard ratio [aHR] 1.53, 95 % CI, 0.766–3.10, P = 0.229). Hyperkalemia is not only responsible for cardiac arrest but also closely correlated with the duration of the cardiac arrest. Therefore, to consider variations in potassium levels based on the timing of cardiac arrest, an additional analysis was conducted, taking into account the duration from the onset of cardiac arrest to arrival at the hospital in individuals with witnessed cardiac arrest whose exact time of cardiac arrest was known (Supplementary Table C). Within this subgroup analysis, no observed correlation was found between the use of anti-hyperkalemic drugs and the ROSC rate in any of the potassium level groups (all P > 0.05).

**Table 3:** Univariable and multivariable Cox regression analyses predicting ROSC following administration of anti-hyperkalemic medications.

	Unadjusted HR	95 % CI	P	Adjusted HR	95 % CI	P
Overall group (N = 838)	0.70	0.58–0.85	<0.001	0.66	0.54–0.81	<0.001
K ≥ 6.5 mmol/L (N = 382)	1.05	0.73–1.53	0.781	0.96	0.65–1.41	0.823
K ≥ 7.5 mmol/L (N = 249)	1.43	0.82–2.49	0.210	1.25	0.69–2.24	0.461
K ≥ 8.5 mmol/L (N = 153)	1.91	0.80–4.56	0.147	1.92	0.73–5.04	0.187

## 4 Discussion

Cardiac arrest is a condition in which circulation is severely impaired, and even with high-quality CPR performed by trained rescuers, cardiac output is only 10–30 % of normal [27,28]. As a result of the patient's drastically altered metabolism, medications administered during cardiac arrest may have different effects than when they are used under normal circumstances. In most patients who survive cardiac arrest, spontaneous circulation is restored within 20–30 min of CPR, but the likelihood of resuscitation decreases significantly if cardiac arrest persists [[29], [30], [31]]. Because of these factors, it is difficult to determine whether the medication administered during CPR has the desired impact within a limited period of time. The purpose of this study was to evaluate whether anti-hyperkalemic agents administered during CPR were effective at lowering serum potassium levels and if they were associated with an improved clinical outcome.

Our main finding in this study was that the administration of anti-hyperkalemic agents during CPR for OHCA did lower serum potassium levels to a greater extent than in cases when these agents were not used, but this did not affect achievement of ROSC nor improve clinical outcomes. Our findings support those of prior studies that reported that neither calcium nor sodium bicarbonate improved the clinical outcomes of cardiac arrest victims [21,[23], [24], [25], [26]]. Existing studies, however, have focused on medications routinely administered to cardiac arrest patients or used in situations of protracted cardiac arrest, and more evidence regarding the use of anti-hyperkalemic agents in cardiac arrest with hyperkalemia is required.

Hyperkalemia is a potentially dangerous electrolyte imbalance that may result in cardiac arrest. In a recent multicenter study, elevated serum potassium levels were associated with worse outcomes in OHCA patients [10]. Therefore, addressing hyperkalemia appropriately during cardiopulmonary resuscitation may have the potential to improve patient prognosis. In this study, the use of anti-hyperkalemic agents during CPR was associated with a decrease in potassium levels. In the anti-hyperkalemic group, the initial potassium level was significantly higher, and the ROSC rate was substantially lower, so we determined that hyperkalemia itself had an effect on the outcome rather than the effect of hyperkalemia medication. As a result, a sub-analysis was conducted in accordance with the potassium concentration. Nonetheless, the administration of anti-hyperkalemic agents did not significantly affect the ROSC rate in any subgroups categorized by the degree of hyperkalemia.

Giving calcium and sodium bicarbonate to patients with severe hyperkalemia improved their clinical prognosis in a previous study of in-hospital cardiac arrest patients [22]. We feel this is a similar pattern to that seen in this study, where the hazard ratios for ROSC increased in the group with higher blood potassium levels when anti-hyperkalemic drugs were used. Our research is noteworthy because we examined the efficacy of anti-hyperkalemic medicines at reducing blood potassium levels in out-of-hospital cardiac arrest patients as well as the influence of these lowered potassium levels on clinical outcomes. The effectiveness of anti-hyperkalemic medicines in cardiac arrest with hyperkalemia requires more investigation.

The following are some of the limitations of the study. First, because this study was performed in a single center, our findings may not be generalizable to other settings. In addition, the power of this investigation is limited by the relatively small sample size. Second, because this was a retrospective observational study with no intervention, selection bias is possible. Third, in the majority of our data, we were only able to verify the administration of anti-hyperkalemic drugs during the CPR process but were unable to access accurate records regarding the precise timing of drug administration. Hence, we were unable to evaluate the impact of drug administration time. Fourth, a substantial number of patients were omitted from the analysis because their potassium concentrations were never measured during CPR, most likely due to the short CPR time, which may have introduced bias. Fifth, the first ROSC was the study's primary endpoint. When determining a patient's actual prognosis, ROSC may be less important than survival discharge and neurologic outcome. However, the purpose of this study was to determine whether causes of fatal and correctable cardiac arrest could be effectively managed during resuscitation. If a correctable cause is not treated effectively, the patient's chances of recovering spontaneous circulation are drastically diminished. Therefore, we determined that the ROSC was a more appropriate primary endpoint for evaluating the effect of therapeutic drugs on hyperkalemia during CPR. In cases of repeated cardiac arrest, the first ROSC was chosen as the endpoint because we believed that evaluating the effects of drugs and blood potassium levels due to physiological changes in the state of spontaneous circulation would involve too many variables. Sixth, it is conceivable that patients with heterogeneous conditions, such as calcium blocker overdose and severe metabolic acidosis, were included in this study. Seventh, we did not comprehensively account for potential confounders, such as the time interval between the occurrence of cardiac arrest and the initiation of resuscitation or the delay in transportation. Additionally, even though we performed adjustments for the initial potassium concentration, we may have failed to make enough adjustments for the impact of hyperkalemia on ROSC. Similar to other retrospective studies, we did not account for variables such as CPR team skill or teamwork, or quality of chest compressions.

## 5 Conclusions

Anti-hyperkalemic agents significantly reduced potassium levels in OHCA patients but did not alter their clinical prognosis. The effectiveness of anti-hyperkalemic agents in CPR for OHCA patients with severe hyperkalemia warrants further study.

## Availability of data and materials

The data and materials used in the current study are all available from the corresponding author upon reasonable request.

## Funding

This work was supported by a 10.13039/501100003725National Research Foundation of Korea grant (NRF-2020R1F1A1075904) funded by the Korean government**.**

## Previous presentations

None.

## Ethics approval and consent to participate

The study was approved by the Institutional Review Board of Samsung Medical Center (IRB No.: 2022-04-130). The need for informed consent was waived given the study's retrospective, observational, and anonymous nature.

## CRediT authorship contribution statement

**Gun Tak Lee:** Writing – original draft, Software, Conceptualization. **Daun Jeong:** Methodology, Investigation. **Jong Eun Park:** Methodology. **Se Uk Lee:** Data curation. **Taerim Kim:** Investigation. **Hee Yoon:** Data curation. **Won Chul Cha:** Investigation. **Min Seob Sim:** Validation, Software. **Ik Joon Jo:** Supervision. **Sung Yeon Hwang:** Writing – review & editing, Supervision. **Tae Gun Shin:** Writing – review & editing, Conceptualization.

## Declaration of competing interest

The authors declare the following financial interests/personal relationships which may be considered as potential competing interests:Tae Gun Shin reports financial support was provided by 10.13039/501100003725National Research Foundation of Korea. If there are other authors, they declare that they have no known competing financial interests or personal relationships that could have appeared to influence the work reported in this paper.
