# Supplementary figures and images for: Efficacy of anti-hyperkalemic agents during cardiopulmonary resuscitation in out-of-hospital cardiac arrest
Source: Heliyon. 2024 Aug 15;10(16):e36345. doi: 10.1016/j.heliyon.2024.e36345 (PMC11381597; doi:10.1016/j.heliyon.2024.e36345)

## Slide 1
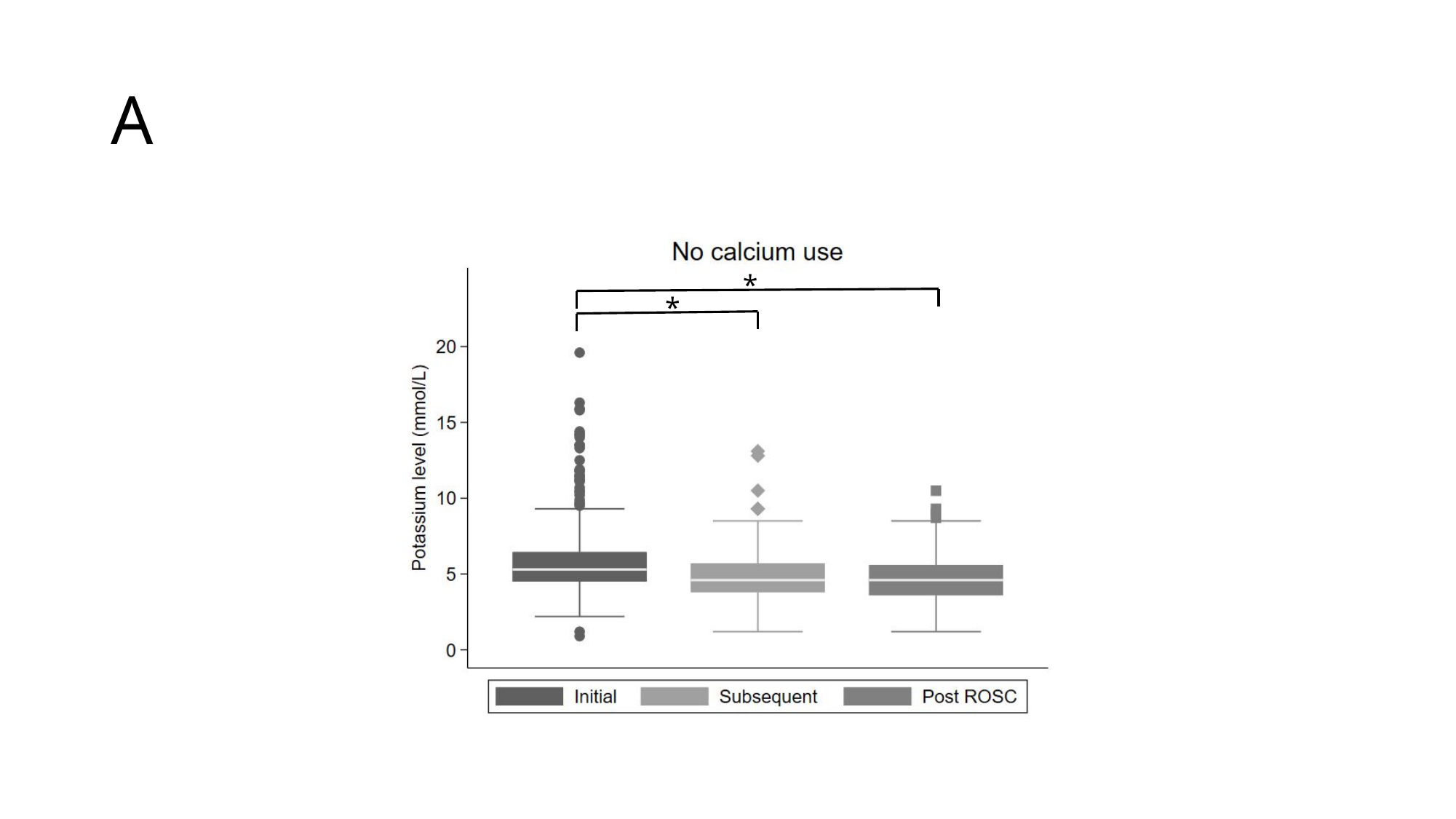

# A
*
*

## Slide 2
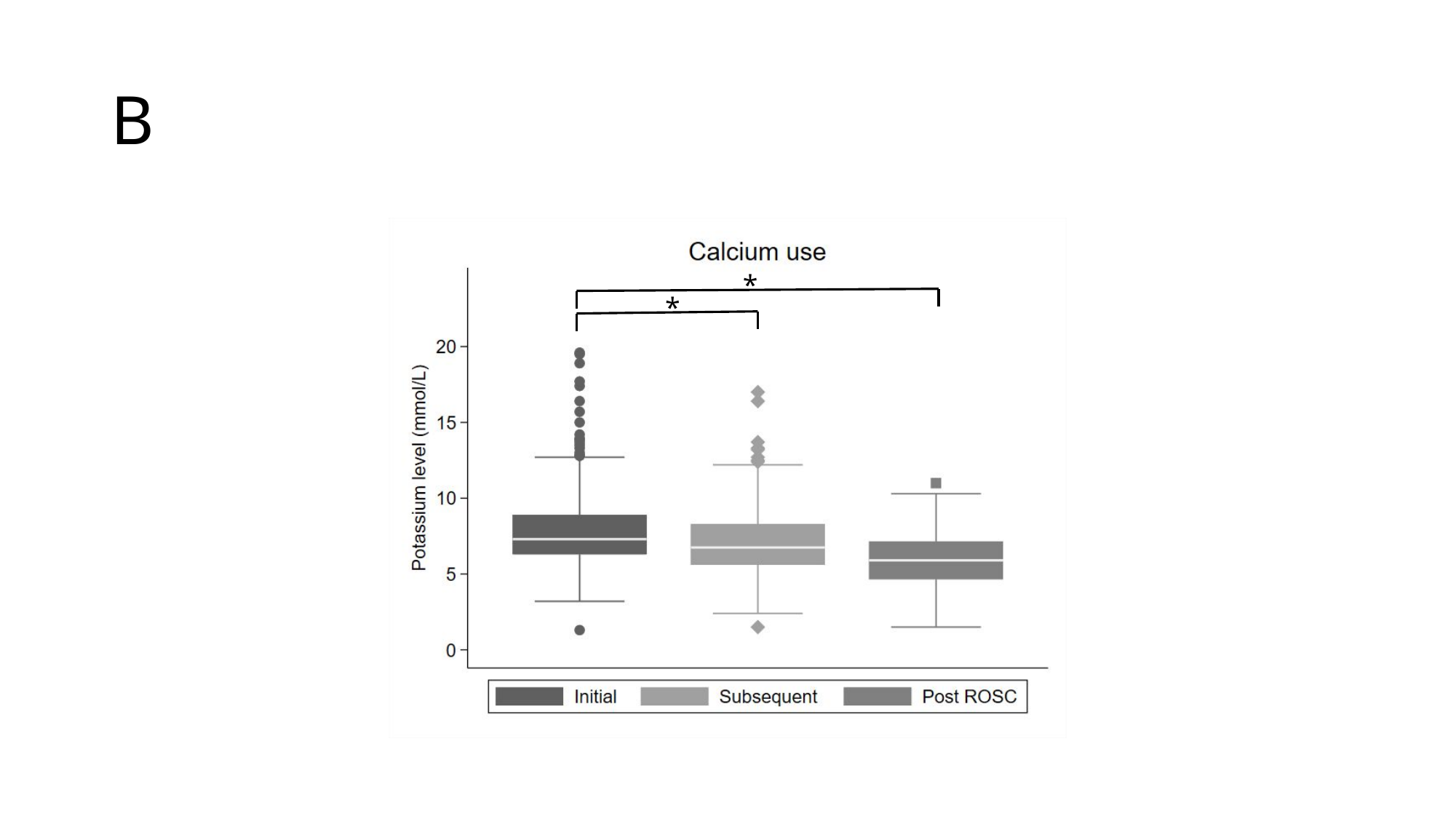

# B
*
*

## Slide 3
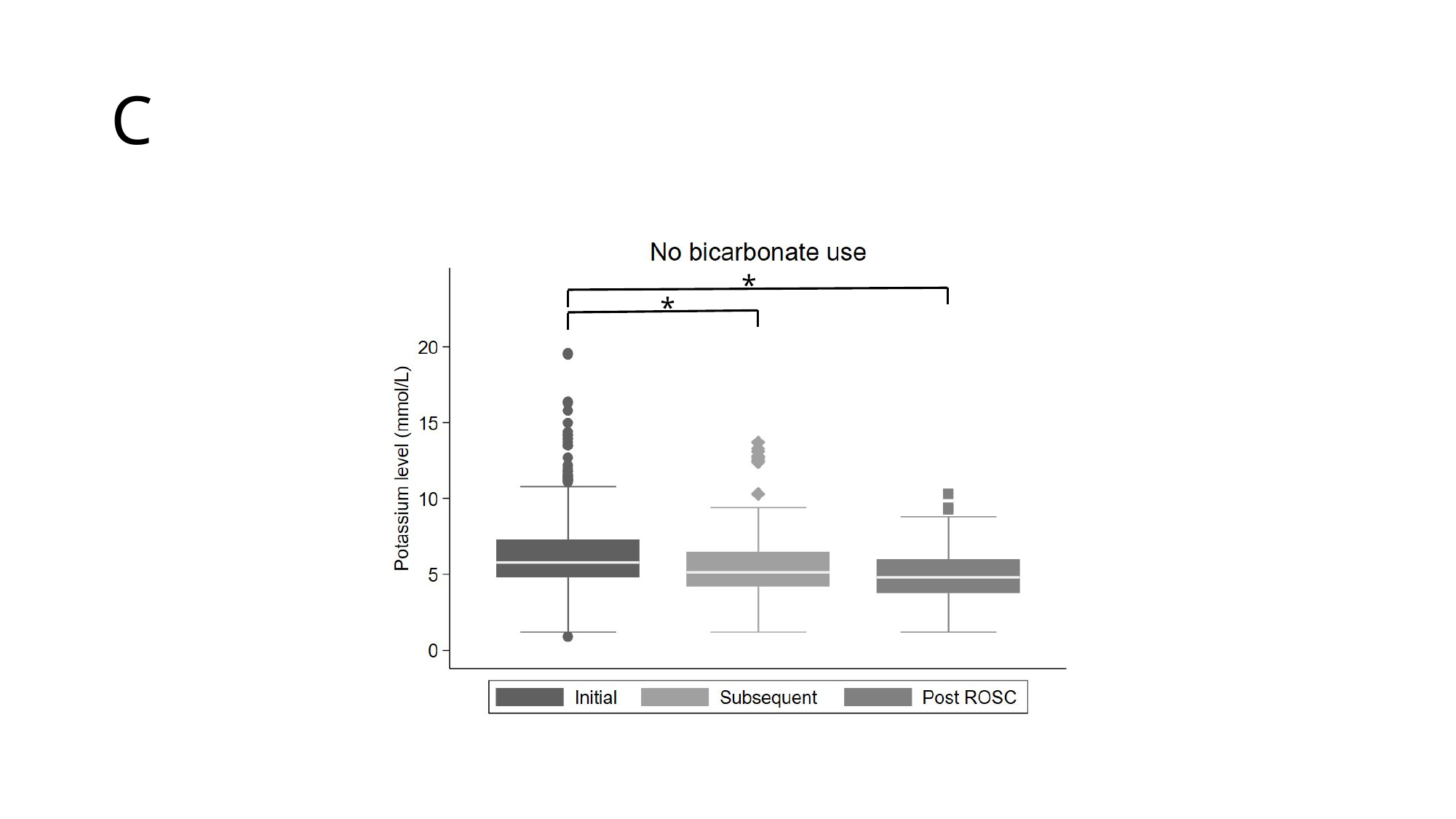

# C
*
*

## Slide 4
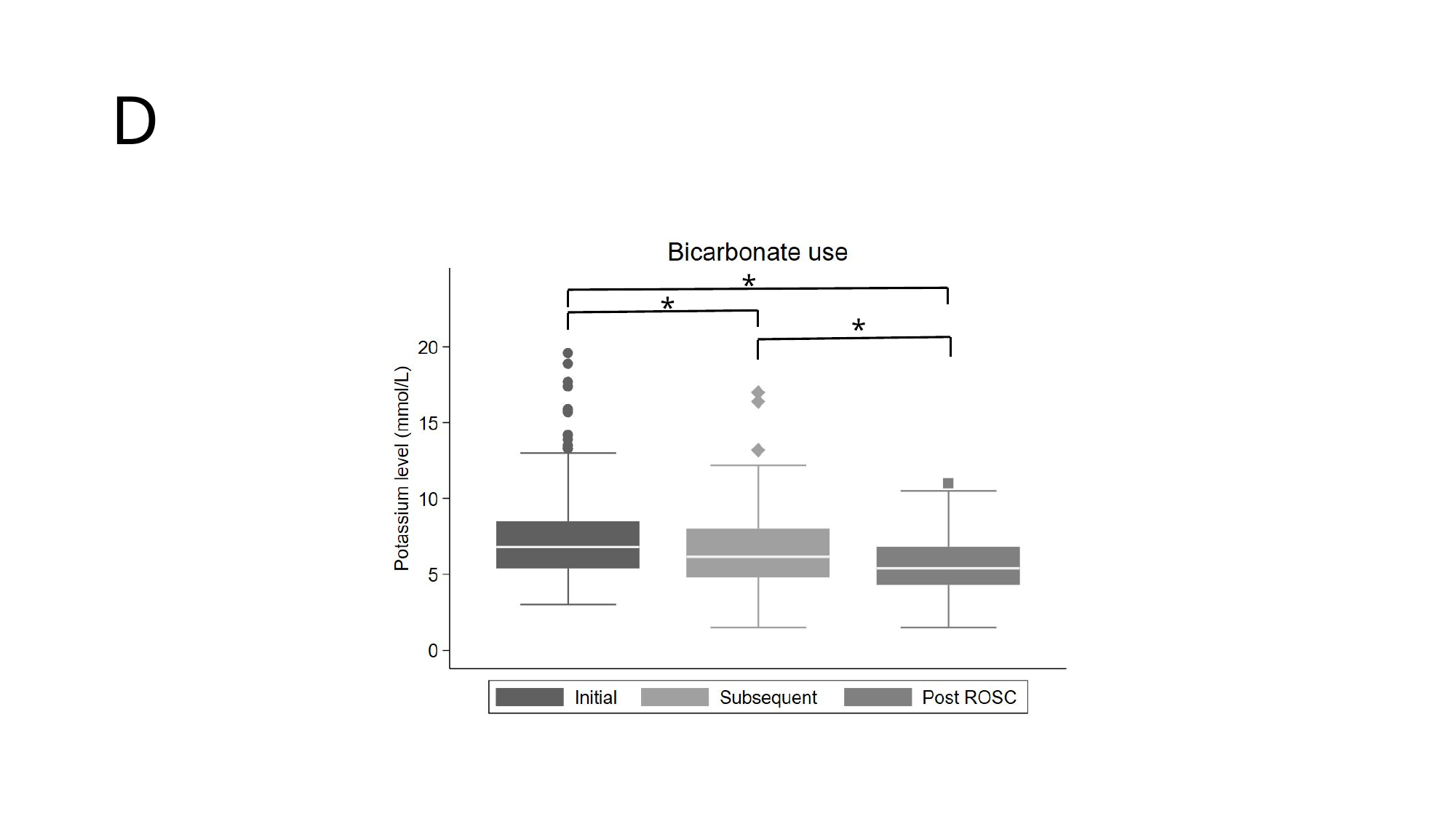

# D
*
*
*

## Slide 5
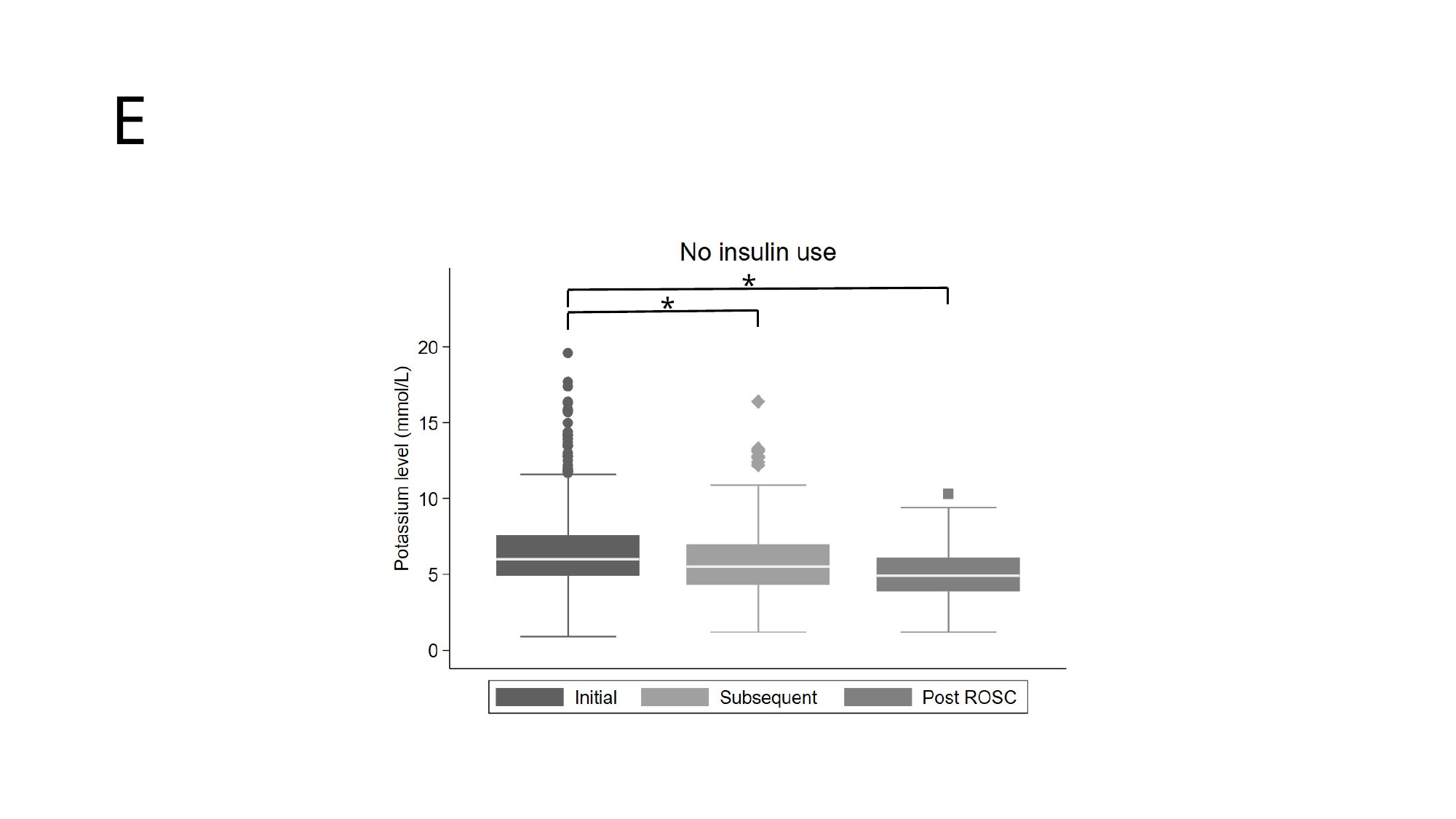

# E
*
*

## Slide 6
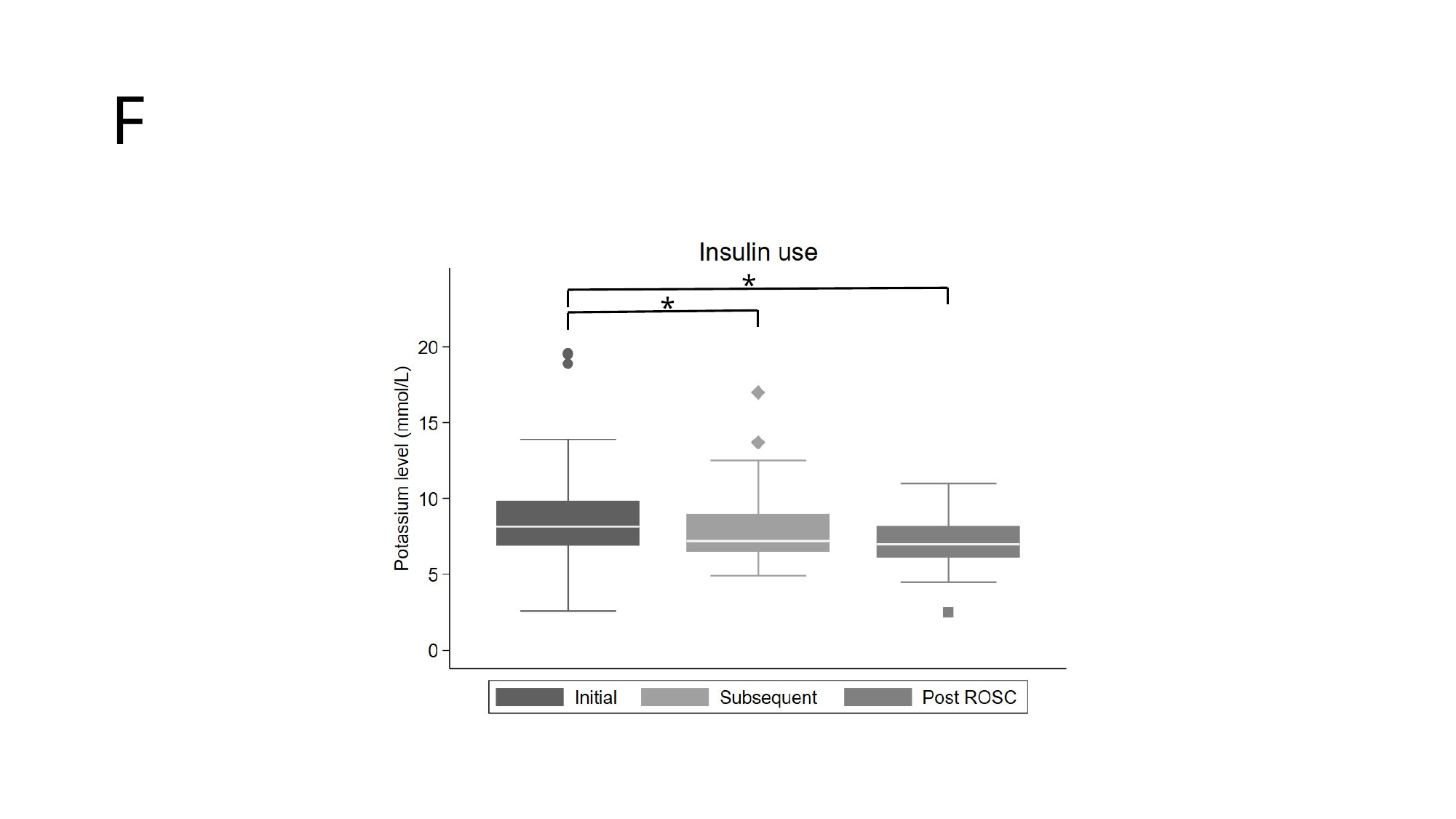

# F
*
*

Supplement: Multimedia component 1 [file mmc1.pptx]
