# Supplementary material for: Efficacy of anti-hyperkalemic agents during cardiopulmonary resuscitation in out-of-hospital cardiac arrest
Source: Heliyon. 2024 Aug 15;10(16):e36345. doi: 10.1016/j.heliyon.2024.e36345 (PMC11381597; doi:10.1016/j.heliyon.2024.e36345)
Supplement: Multimedia component 2 [file mmc2.docx]

|  | Overall | No anti-hyperkalemic | Anti-hyperkalemic | P |
| --- | --- | --- | --- | --- |
| Initial K level (mmol/L) | 6.2 (5.0–7.8)  (n=839) | 5.4 (4.6–6.6)  (n=374) | 6.9 (5.7–8.4)  (n=465) | <0.001 |
| Subsequent K level (mmol/L) | 5.7 (4.4–7.2)  (n=318) | 4.7 (3.9–5.7)  (n=102) | 6.3 (5.0–8.0)  (n=216) | <0.001 |
| Post ROSC K level (mmol/L) | 5.1 (4.0–6.3)  (n=374) | 4.5 (3.6–5.7)  (n=183) | 5.4 (4.4–6.8)  (n=193) | <0.001 |

**Supplementary Table A.** Potassium levels.

**Supplementary Table B.** Linear regression analysis of the change in potassium levels.

|  | Coefficient | 95% CI | P |
| --- | --- | --- | --- |
| Delta K (initial to subsequent levels during CPR) | 0.21 | -0.14-0.56 | 0.234 |
| Delta K (initial to post-ROSC levels) | 0.38 | 0.13-0.64 | 0.003 |

All results are for the anti-hyperkalemic group compared to the no anti-hyperkalemic group.

**Supplementary Table C.** Multivariate Cox regression analysis predicting ROSC after antihyperkalemic drug administration considering cardiac arrest duration.

|  | Adjusted HR | 95% CI | P |
| --- | --- | --- | --- |
| K ≥6.5 mmol/L  (N=210) | 0.96 | 0.60–1.54 | 0.858 |
| K ≥7.5 mmol/L  (N=127) | 1.19 | 0.59–2.42 | 0.624 |
| K ≥8.5 mmol/L  (N=65) | 2.39 | 0.54–10.64 | 0.254 |

The analysis was conducted, considering the duration from the onset of cardiac arrest to hospital arrival in patients with witnessed cardiac arrest for whom the exact time of cardiac arrest was documented.

Adjusted variables: age, gender, pre-existing diseases (diabetes, malignancy, chronic kidney disease), arrest location, bystander CPR, initial ECG rhythm, initial potassium level, duration from onset of cardiac arrest to hospital arrival

**Figure legends**

Supplementary figure 1. Initial, subsequent, and post ROSC potassium levels with and without the use of each anti-hyperkalemic medications (* P<0.05).
